# Preoperative chronic kidney disease predicts poor oncological outcomes after radical cystectomy in patients with muscle-invasive bladder cancer

**DOI:** 10.18632/oncotarget.18248

**Published:** 2017-05-29

**Authors:** Itsuto Hamano, Shingo Hatakeyama, Hiromichi Iwamurau, Naoki Fujita, Ken Fukushi, Takuma Narita, Kazuhisa Hagiwara, Ayumu Kusaka, Shogo Hosogoe, Hayato Yamamoto, Yuki Tobisawa, Tohru Yoneyama, Takahiro Yoneyama, Yasuhiro Hashimoto, Takuya Koie, Hiroyuki Ito, Kazuaki Yoshikawa, Toshiaki Kawaguchi, Chikara Ohyama

**Affiliations:** ^1^ Department of Urology, Hirosaki University Graduate School of Medicine, Hirosaki, Japan; ^2^ Department of Advanced Transplant and Regenerative Medicine, Hirosaki University Graduate School of Medicine, Hirosaki, Japan; ^3^ Department of Urology, Aomori Rosai Hospital, Hachinohe, Japan; ^4^ Department of Urology, Mutsu General Hospital, Mutsu, Japan; ^5^ Department of Urology, Aomori Prefectural Central Hospital, Aomori, Japan

**Keywords:** bladder cancer, radical cystectomy, survival, chronic kidney disease, renal function

## Abstract

**Objective:**

To evaluate the impact of preoperative chronic kidney disease (CKD) on oncologic outcomes in muscle-invasive bladder cancer patients who underwent radical cystectomy.

**Methods:**

A total of 581 patients who underwent radical cystectomy at four medical centers between January 1995 and February 2017 were examined retrospectively. We investigated oncologic outcomes, including progression-free, cancer-specific, and overall survival (PFS, CSS, and OS, respectively) stratified by preoperative CKD status (pre-CKD vs. non-CKD). We performed a Cox proportional hazards regression analysis using inverse probability of treatment weighting (IPTW) to evaluate the impact of preoperative CKD on prognosis and developed the prognostic factor-based risk stratification nomogram

**Results:**

Of the 581 patients, 215 (37%) were diagnosed with CKD before radical cystectomy. Before the background adjustment, PFS, CSS, and OS after radical cystectomy were significantly lower in the pre-CKD group compared to the non-CKD group. Background-adjusted IPTW analysis showed that preoperative CKD was significantly associated with poor PFS, CSS, and OS after radical cystectomy. The nomogram for predicting 5-year PFS and OS probability showed significant correlation with actual PFS and OS (*c*-index = 0.73 and 0.77, respectively).

**Conclusions:**

Muscle-invasive bladder cancer patients with preoperative CKD had a significantly lower survival probability than those without CKD.

## INTRODUCTION

Bladder cancer is the 11^th^ most commonly diagnosed cancer and the 14^th^ leading cause of cancer deaths worldwide [1]. Radical cystectomy (RC) with extended pelvic lymph node dissection is the standard treatment for nonmetastatic muscle-invasive bladder cancer (MIBC) [2, 3]. Long-term outcomes and predictors of disease relapse after RC are well documented. Performance status, old age, tobacco smoking, waiting time for surgery, tumor stage, lymphovascular invasion (LVI), lymph node involvement, and symptomatic recurrence are regarded as significant prognostic risk factors [2–7].

Bladder cancer is a disease of middle-aged and elderly people. As the population is aging, bladder cancer will occur more frequently in the near future [8]. Chronic kidney disease (CKD) is common in elderly patients with bladder cancer [9–12]. In addition, increasing evidence has suggested that preoperative renal insufficiency indicates poor prognosis in upper urinary tract carcinoma [13, 14], nonmuscle-invasive bladder cancer [15], and MIBC [14, 16, 17]. Although several studies have investigated postoperative CKD in MIBC patients with urinary diversion [2, 3, 11, 18], few studies have evaluated the direct influence of preoperative CKD on oncologic prognosis in MIBC patients after RC. Because a prospective randomized study is not feasible, statistical methods must be used to remove the effects of confounding factors due to nonrandom treatment assignment in retrospective studies. We compared oncologic outcomes between MIBC patients with and without preoperative CKD using inverse probability of treatment weighting (IPTW) via the propensity score and developed a prognostic factor-based risk stratification nomogram.

## RESULTS

### Baseline characteristics

Of the 581 patients, 215 (37%) were diagnosed with pre-CKD (estimated glomerular filtration rate [eGFR] <60 mL/min/1.73 m2) before RC. The disease recurred in 175 patients (30%). There were significant differences in patient characteristics between the groups in terms of age (*P* < 0.001), cardiovascular disease (CVD; *P* = 0.018), preoperative eGFR (*P* < 0.001), number of patients who underwent neoadjuvant chemotherapy (NAC) (*P* = 0.021), orthotopic neobladder construction (*P* < 0.001), and disease stage ≥pT3 (*P* = 0.023; Table 1).

**Table 1:** Background of patients

	Non-CKD	Pre-CKD	*P* value
n	366	215	
Age, years	66±9.4	71±7.9	*<0.001*
Male, n=	295 (81%)	163 (76%)	*0.173*
ECOG PS >0, n=	8 (2.2%)	8 (3.3%)	*0.432*
Hypertension (HTN), n=	104 (28%)	75 (35%)	*0.103*
Cardiovascular disease (CVD), n=	34 (9.3%)	34 (16%)	*0.018*
Diabetes mellitus (DM), n=	51 (14%)	27 (13%)	*0.638*
Pre-operative eGFR	77±13	46±11	*<0.001*
CKD stage 3A, n=		138 (64%)	
≥CKD stage 3B, n=		77 (36%)	
Neoadjuvant chemotherapy (NAC), n=	233 (64%)	116 (54%)	*0.021*
Clinical staging			
Stage ≥cT3	174 (48%)	102 (47%)	*0.982*
cN+	33 (9.0%)	25 (12%)	*0.311*
Surgical outcomes			
Robotic surgery, n=	18 (4.9%)	5 (2.3%)	*0.122*
Urinary diversion (neobladder), n=	228 (62%)	87 (40%)	*<0.001*
Post-operative complications (≥Grade 3)	9 (2.5%)	8 (3.7%)	*0.384*
Pathological outcomes			
Tumor grade (High), n=	245 (67%)	151 (70%)	*0.411*
Stage ≥pT3, n=	103 (28%)	80 (37%)	*0.023*
Lymphovascular invasion (LVI+), n=	117 (32%)	77 (36%)	*0.342*
pN+, n=	41 (11%)	29 (13%)	*0.414*
Oncological outcomes			
Tumor recurrence, n=	97 (26%)	81 (38%)	*0.002*
Cancer mortality, n=	68 (19%)	70 (33%)	*<0.001*
Overall mortality, n=	100 (27%)	89 (41%)	*<0.001*

CKD: chronic kidney disease.

### Oncological outcomes

**Figure 1:**
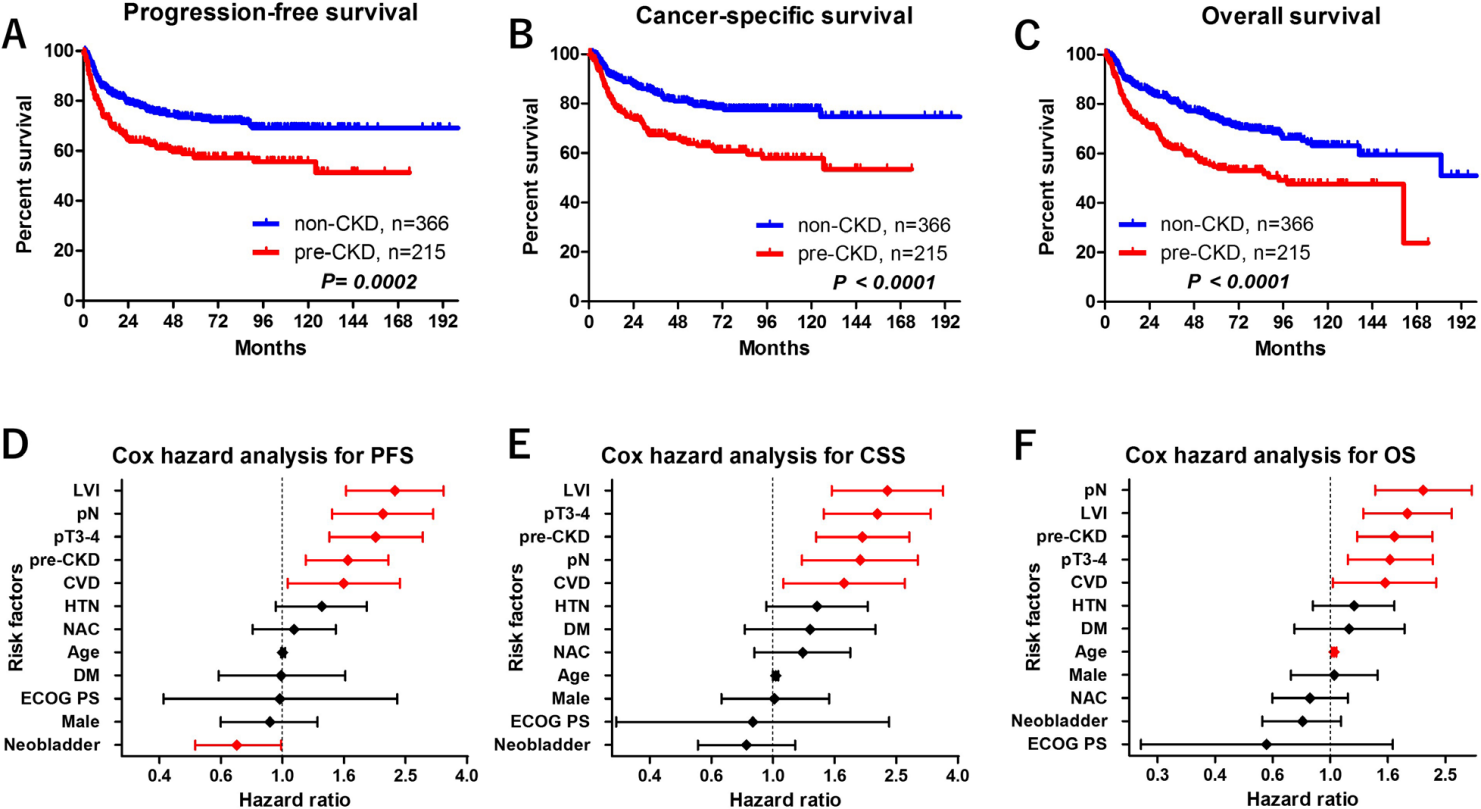
Oncological outcomes Before the background adjustment, there were statistically significant differences in PFS **(A)**, CSS **(B)**, and OS **(C)** between the groups. The pre-CKD group had significantly lower 5-year PFS (57% vs. 73%, *P* = 0.002), 5-year CSS (63% vs. 79%, *P* < 0.001), and 5-year OS (55% vs. 74%, *P* < 0.001) compared to the non-CKD group. In multivariate Cox proportional hazards regression analysis, LVI, pN, stage ≥pT3, pre-CKD, and CVD were selected as independent predictors for PFS **(D)** and CSS **(E)**. Neobladder was selected as a independent factor for PFS. Similarly, pN, LVI, CVD, pre-CKD, stage ≥pT3, and age were selected as independent predictors for OS **(F)**.

Median follow-up periods in the pre-CKD and non-CKD groups were 32 and 54 months, respectively. Before the background adjustment, there were significant differences in the number of patients experiencing tumor recurrence (*P* = 0.002), cancer mortality (*P* < 0.001), and overall mortality (*P* < 0.001) in the pre-CKD compared with the non-CKD groups (Table 1). There were statistically significant differences in the progression-free, cancer-specific, and overall survival (PFS, CSS, and OS, respectively) measures between the groups (Figure 1A–1C). The pre-CKD group had significantly worse oncologic outcomes than the non-CKD group. Five-year PFS, CSS, and OS rates for the pre-CKD and non-CKD groups were 57% vs. 73% (*P* = 0.002), 63% vs. 79% (*P* < 0.001), and 55% vs. 74% (*P* < 0.001), respectively.

In multivariate Cox proportional hazards regression analysis, LVI, pN, stage ≥pT3, pre-CKD, and CVD were selected as independent predictors for PFS (Figure 1D) and CSS (Figure 1E). Similarly, pN, LVI, CVD, pre-CKD, stage ≥pT3, and age were selected as independent predictors for OS (Figure 1F, Table 2).

**Table 2:** Multivariate Cox regression analysis for prognosis

	Risk factor	PFS			CSS			OS		
		*P* value	HR	95%CI	*P* value	HR	95%CI	*P* value	HR	95%CI
Age	Continuous	*0.640*	1.00	0.99-1.02	*0.115*	1.02	1.00-1.04	*0.001*	1.03	1.01-1.05
Sex	Male	*0.591*	0.91	0.63-1.30	*0.947*	1.01	0.68-1.52	*0.891*	1.02	0.73-1.45
ECOG PS	>0	*0.968*	0.98	0.41-2.36	*0.864*	0.31	0.31-2.38	*0.502*	0.73	0.30-1.81
HTN	Positive	*0.097*	1.34	0.95-1.88	*0.090*	1.39	0.95-2.03	*0.256*	1.21	0.87-1.67
DM	Positive	*0.978*	0.99	0.62-1.60	*0.270*	1.32	0.81-2.15	*0.604*	1.12	0.72-1.75
CVD	Positive	*0.034*	1.58	1.04-2.41	*0.023*	1.70	1.08-2.68	*0.022*	1.61	1.07-2.41
Renal function	Pre-CKD	*0.002*	1.63	1.19-2.21	*<0.001*	1.95	1.38-2.77	*0.001*	1.67	1.24-2.25
NAC	Underwent	*0.579*	1.09	0.80-1.49	*0.225*	1.25	0.87-1.78	*0.366*	0.87	0.64-1.18
Urinary diversion	Neobladder	*0.041*	0.71	0.52-0.99	*0.273*	0.82	0.57-1.18	*0.147*	0.79	0.58-1.09
pT	≥3	*<0.001*	2.01	1.42-2.86	*<0.001*	2.18	1.46-3.25	*<0.001*	1.90	1.34-2.70
pN	Positive	*<0.001*	2.12	1.45-3.09	*<0.001*	1.92	1.24-2.95	*<0.001*	2.09	1.42-3.07
LVI	Positive	*<0.001*	2.32	1.61-3.34	*<0.001*	2.35	1.55-3.56	*0.011*	1.55	1.11-2.18

ECOG PS: Eastern Cooperative Oncology Group performance status; HTN: hypertension; CVD: cardiovascular disease; DM: diabetes mellitus; CKD: chronic kidney disease; NAC: neoadjuvant chemotherapy; LVI: lymphovascular invasion; PFS: progression-free survival; CSS: cancer-specific survival; OS: overall survival; HR: hazard ratio.

### IPTW analyses for PFS, CSS, and OS

Background-adjusted IPTW analysis showed that preoperative CKD was significantly associated with poor PFS (*P* = 0.046; hazard ratio [HR], 1.39; 95% confidence interval [CI], 1.01–1.92), CSS (*P* = 0.002; HR, 1.74; 95% CI, 1.21–2.48), and OS (*P* = 0.022; HR, 1.43; 95% CI, 1.05–1.95) after RC (Table 3).

**Table 3:** Cox proportional hazard regression analysis using IPTW* for prognosis

	*P* value	HR	95%CI
Progression-free survival (PFS)	*0.046*	1.39	1.01-1.92
Cancer-specific survival (CSS)	*0.002*	1.74	1.21-2.48
Overall survival (OS)	*0.022*	1.43	1.05-1.95

*, Inverse probability of treatment weighting. Variables for adjustment: age, sex, ECOG PS, HTN, CVD, DM, NAC, urinary diversion, LVI, stage ≥pT3, pN.

### The nomogram for 5-year PFS and OS probability

**Figure 2:**
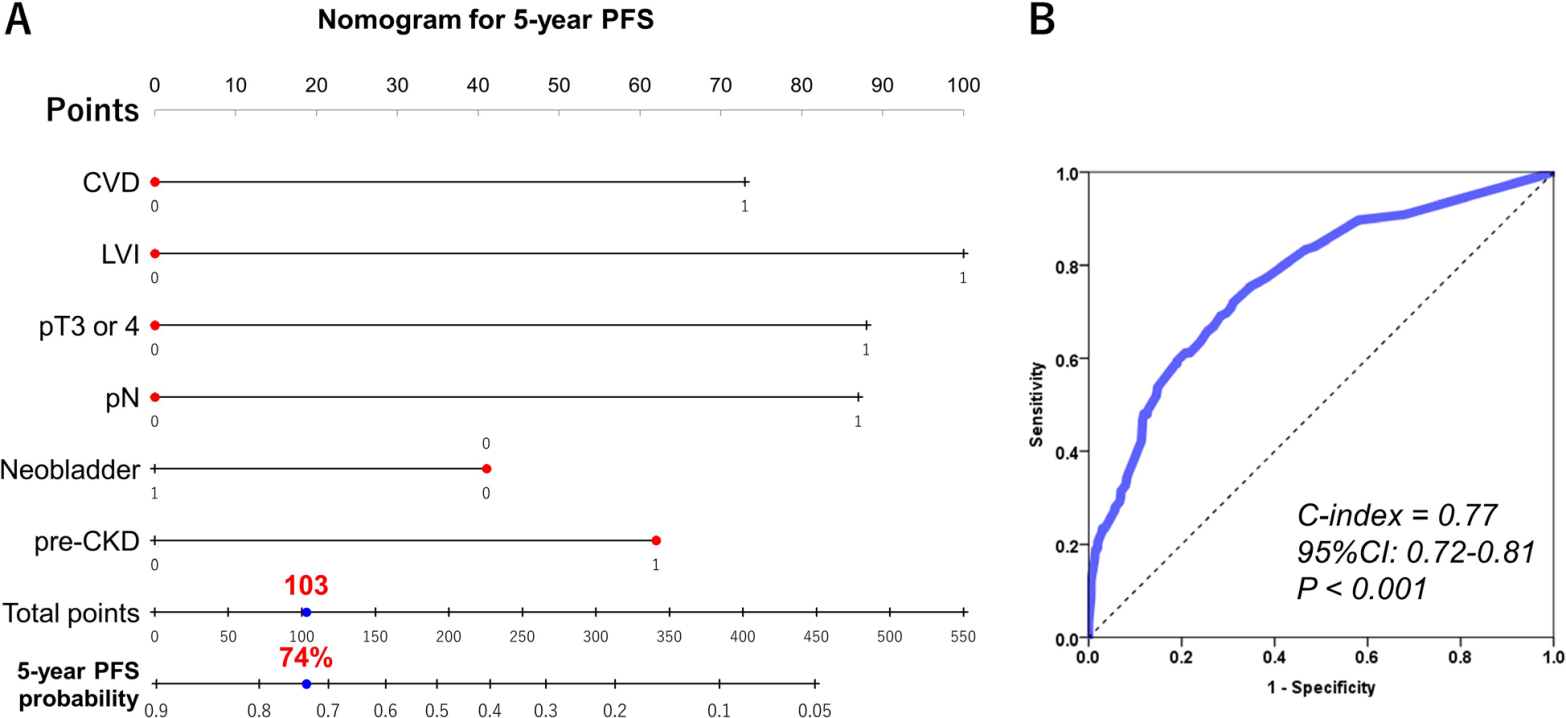
Predictive model for five-year progression-free survival The nomogram including CVD, pre-CKD, neobladder, LVI, stage ≥pT3, and pN for predicting five-year PFS is shown **(A)**. The calculation for 5-year PFS probability in the case of pre-CKD patients who underwent neobladder substitution without CVD, LVI, stage ≥pT3, and pN provided a value of 74%. The nomogram showed significant correlation between estimated and actual OS (c-index = 0.77, *P* < 0.001, 95% CI: 0.72–0.81) **(B)**.

**Figure 3:**
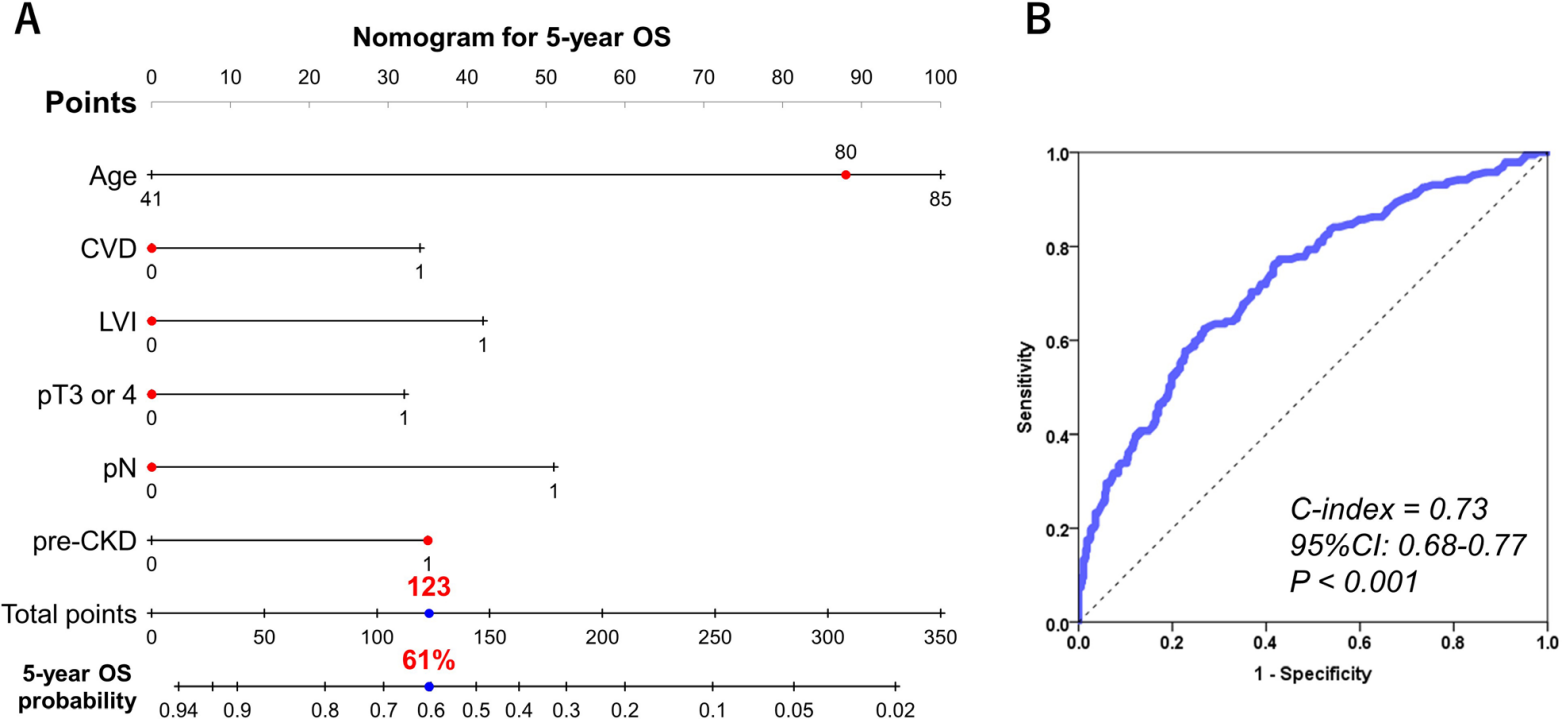
Predictive model for 5-year overall survival The nomogram including age, CVD, pre-CKD, LVI, stage ≥pT3, and pN for predicting 5-year OS is shown **(A)**. The calculation for 5-year OS probability in the case of 80-year-old pre-CKD patients without CVD, LVI, stage ≥pT3, and pN provided a value of 61%. The nomogram showed significant correlation between estimated and actual OS (c-index = 0.73; *P* < 0.001; 95% CI, 0.68–0.77) **(B)**.

Independent predictors, including neobladder, CVD, pre-CKD, LVI, stage ≥pT3, and pN were used to develop the nomogram predicting 5-year PFS (Figure 2A). This model revealed significant correlation between estimated and actual PFS (Figure 2B) (*c*-index = 0.77, *P* < 0.001, 95% CI: 0.72–0.81). Independent predictors, including age, CVD, pre-CKD, LVI, stage ≥pT3, and pN were used to develop the nomogram predicting 5-year OS (Figure 3A). This model revealed significant correlation between estimated and actual OS (Figure 3B; *c*-index = 0.73; *P* < 0.001; 95% CI, 0.68–0.77). The risk calculations for PFS and OS are provided in a Supplementary File 1 (MS Excel, File 1).

## DISCUSSION

Interest in the influence of preoperative renal insufficiency on cancer prognosis has increased because of its prevalence in elderly patients with MIBC. Our results demonstrated the prevalence of preoperative CKD to be 37%, and it was an independent predictor of PFS, CSS, and OS in patients with MIBC who underwent RC. The independent predictive value of preoperative CKD persisted after multivariable Cox proportional hazards regression analysis accounting for established prognostic factors, such as pN+, LVI+, stage ≥pT3, CVD, and age. In addition, background-adjusted IPTW analyses selected preoperative CKD as a significant factor for prognosis.

Although a cause-and-effect relationship between renal insufficiency and cancer has not been established, evidence from a prospective population-based cohort study of 3,654 residents at the New South Wales Cancer Registry supports our findings that incidences of malignancies (lung and urinary tract) increased in patients with CKD [19]. Previously, Li CE et al. found that patients with CKD had a worse prognosis, higher tumor recurrence and progression rates in primary nonmuscle-invasive bladder cancer [15]. Moreover, they suggested that declined eGFR (eGFR <30 mL/min/1.73m2) was also associated with higher recurrence risk and poorer overall survival in patients with CKD than in those without CKD [15]. However, few recent studies have documented poor oncologic outcomes using CKD (eGFR <60 mL/min/1.73 m2) criteria in MIBC patients who underwent RC [16, 20]. Thrasher et al. demonstrated that T-stage (≥cT2), older age (≥65 years), higher tumor grade, lower preoperative hemoglobin level (≤12 g/dL), and higher preoperative creatinine level (≥1.5 mg/dL) were predictive of poor CSS after RC in 531 patients with MIBC [16]. In the present study, age, history of CVD, presence of preoperative CKD, stage ≥pT3, LVI+, and pN+ were identified as prognostic factors for OS. Our nomogram for predicting 5-year OS showed significant correlation between the estimated and actual OS (*c*-index = 0.73, *P* < 0.001). As shown in Figure 3A and the Supplementary File 1 (MS Excel, File 1), preoperative CKD decreased 5-year OS probability from 76% to 61% (a 15% decline) in 80-year-old MIBC patients without CVD, LVI, stage ≥pT3, and pN. These results suggest that the impact of preoperative CKD cannot be ignored. A recent meta-analysis [14] has suggested that the prevalence of preoperative renal insufficiency with bladder cancer was 16.9% (ranging from 13.0%–25.5%) and that preoperative renal insufficiency was associated with increased disease recurrence (HR = 1.65; 95% CI, 1.11–2.19), CSS (HR = 1.59; 95% CI, 1.14–2.05), and OS (HR = 1.45; 95% CI, 1.19–1.71). However, the limitation of this meta-analysis which included 16 retrospective studies was the lack of a universal testing method (either serum creatinine or eGFR was used to indicate renal function) and classification of renal insufficiency between the studies. Therefore, direct evidence for the relationship between preoperative CKD and the oncologic outcome is needed.

Our results showed a strong correlation between preoperative CKD and poor prognosis, suggesting that the CKD patients with urothelial carcinoma might have aggressive cancer behavior leading to disease progression and recurrence. In general, CKD is strongly associated with all-cause mortality, especially in cardiovascular-related mortality [21]. Therefore, it is not surprising that the number of patients with CVD was higher in the pre-CKD group in the present study (Table 1). However, the reason for the strong association between CKD and cancer recurrence and mortality remains undetermined. Although there is no clear explanation, the effects of metabolic syndrome [22] and chronic inflammation may explain the association of CKD and oncological outcome [21, 23]. Immunocompromised CKD patients may have reduced DNA repair capacity and protection against viral oncogene [24]. Uremia-associated immune deficiency is a well-known complication of loss of renal function. Chronic inflammation and increased oxidative stress underlie the uremia-associated immune deficiency [25]. Long-term inflammation and oxidative stress caused by CKD and linked to organ degradation may increase carcinogenicity. The other potential factor that can result in a poor prognosis is frailty. Patients with CKD and CVD are more likely to be frail [26] because these diseases are likely to exist in combination with comorbid conditions, disability, and polypharmacy [27, 28]. In addition, frailty is also one of the important parameters of cancers [29, 30]. A recent study reported the association between frailty and inflammatory markers in elderly cancer patients [31]. Our previous study suggested that renal function has potential to predict postoperative frailty [32]. Although we could not address the direct reason as to how CKD and CVD were independently influencing recurrence after RC, these results suggest a potential relationship between cancer progression, CKD, CVD, and frailty. However, evidence to confirm this hypothesis is lacking. Future studies to assess mechanisms underlying carcinogenesis and CKD are warranted.

The effect of CKD on the risk of poor prognosis with malignant disease is controversial. The presence of CKD was associated with an increased risk of death in cancer patients in several studies [33, 34]. Na SY et al. [33] retrospectively reviewed the cases of 8,223 cancer patients and reported that cancer-specific mortality was significantly higher in the patients with CKD than those without CKD (the adjusted HR: 1.12 for patients with an eGFR of 30-59 mL/min/1.73 m2, *P* = 0.04 and 1.75 for patients with an eGFR <30 mL/min/1.73 m2, *P* < 0.001). However, the impact of CKD on prognosis may differ depending on types of cancers. CKD is not an independent risk factor for survival in patients with lung cancer [33, 35] and breast cancer [33, 36]. Currently, CKD is reported as an independent risk factor for survival in head and neck, stomach, liver, colorectal, urinary tract, gynecological and hematologic malignancy [33, 34].

Because genitourinary cancers are risk factors for renal dysfunction during the disease progress and treatment, preoperative renal insufficiency also serves as a prognostic factor in renal cell carcinoma. Kim YW et al. [37] reported that clinical stage (T and N) and tumor size of renal cell carcinoma were significantly higher and larger, respectively, in patients with CKD than those without CKD in propensity-score-matched cohorts. Recurrence-free survival, CSS, and OS were significantly lower in the patients with CKD. However, the precise biological mechanisms for the association between CKD, types of cancers, and oncological outcomes remain undetermined. Further studies are necessary to determine why CKD has a prognostic impact on selected types of cancers.

Although the clinical standard for the assessment of kidney function is eGFR [38, 39], measurement of creatinine to determine the eGFR has limitations in risk prediction, particularly in patients with reduced muscle mass [40]. Equations based on cystatin C provide an alternative method to estimate eGFR. Several studies have suggested that the addition of cystatin C measurements to creatinine measurements in calculating the eGFR significantly improves the risk classification for death, cardiovascular disease, and end-stage renal disease [41, 42]. However, no study has evaluated the impact of cystatin C on prognosis in bladder cancer patients. Further studies are necessary to investigate the clinical utility of cystatin C in bladder cancer.

Several limitations must be acknowledged. First, the data from multiple centers, the retrospective study design, and patients with short follow-up prevented us from making definitive conclusions regarding the impact of preoperative CKD on prognosis. We could not control the influence of inter-facility difference on prognosis among four hospitals (Supplementary Figure 1). Despite the use of an IPTW method, which is an attractive method for estimating treatment effects using observational data, we were unable to control selection bias and other unmeasurable confounders of retrospective study. Second, an eGFR evaluation using a modified formula for Japanese patients may prevent the generalization of our results to non-Asian populations. In addition, due to the retrospective design, we could not access information regarding preoperative hydronephrosis. Because hydronephrosis is an indicator of the aggressiveness of a tumor, this information should be included in future studies. Third, another validation cohort was needed to verify the accuracy of the nomogram. In addition, a prospective study of the relationship between preoperative CKD and the oncological outcome is necessary. Despite these limitations, we evaluated the direct impact of CKD on oncological outcomes in MIBC patients using IPTW analysis and developed a prognostic factor-based risk stratification nomogram. Our results support the idea that preoperative CKD is an important predictor of not only all-cause mortality but also cancer mortality in patients with MIBC.

In conclusion, MIBC patients with preoperative CKD had a significantly lower survival probability than MIBC patients without CKD after undergoing RC. Further study is needed to assess the impact of renal insufficiency on the prognosis of MIBC.

## MATERIALS AND METHODS

### Design and ethics statement

This retrospective, multicenter study was performed in accordance with the ethical standards of the Declaration of Helsinki and approved by an ethics review board of Hirosaki University School of Medicine (authorization numbers; 2015–258 and 2016–225).

### Patient selection

Between May 1996 and February 2017, 581 adults underwent RC and urinary diversion in Hirosaki University Hospital, Aomori Rosai Hospital, Mutsu General Hospital, and Aomori Prefectural Central Hospital. We stratified the patients into two groups based on preoperative renal function: eGFR ≥60 mL/min/1.73 m2 (non-CKD) and eGFR <60 mL/min/1.73 m2 (pre-CKD).

### Evaluation of variables

The variables analyzed were age, sex, Eastern Cooperative Oncology Group performance status (ECOG PS), clinical stage, renal function before RC, history of hypertension (HTN), CVD, and diabetes mellitus (DM). Renal function was evaluated by eGFR before RC using a modified version of the abbreviated Modification of Diet in Renal Disease Study formula for Japanese patients [43]. Tumor stage and grade were assigned according to the 2009 TNM classification of the Union of International Cancer Control [44]. Postoperative complications were evaluated by Clavien-Dindo classification [45].

### Neoadjuvant chemotherapy (NAC)

Since September 2004, we have performed two or three courses of NAC in MIBC patients. NAC comprised a platinum-based combination regimen, using either gemcitabine plus cisplatin; gemcitabine plus carboplatin; or methotrexate, vinblastine, adriamycin, and cisplatin. Regimens were selected based on guidelines regarding eligibility for the proper use of cisplatin [46] and the patient’s overall status.

### Surgical procedure

All patients underwent RC, urinary diversion, and standard pelvic lymph node dissection (PLND), which included removal of the obturator, external iliac, hypogastric, and common iliac lymph node chains (there were no para-aortic or paracaval dissections). All RCs were performed using the basic technique as we have described previously [47]. An orthotopic ileal neobladder construction, ileal conduit diversion, or cutaneous ureterostomy were performed according to previously reported methods [48–51].

### Patient follow-up

After treatment, each patient was assessed every 3 months via ultrasonography, serum electrolytes, blood urea nitrogen, serum creatinine, and liver function. Computed tomography (CT) was performed every 3–6 months (based on pathologic findings) for the detection of tumor recurrence. Adjuvant chemotherapy was not administered routinely. Salvage therapy was introduced when indicated by CT.

### Outcome evaluations

We retrospectively evaluated pathological T and N stages and LVI in the non-CKD and pre-CKD groups. Oncologic outcomes for both the groups, including PFS, CSS, and OS, were investigated using the Kaplan–Meier method and compared with the log-rank test. Multivariate Cox regression analysis was performed for independent predictors of PFS, CSS, and OS.

### Statistical analysis

Statistical analyses of data were performed using SPSS version 24.0 (SPSS, Inc., Chicago, IL, USA), GraphPad Prism 5.03 (GraphPad Software, San Diego, CA, USA), and R 3.3.2 (The R Foundation for Statistical Computing, Vienna, Austria). Categorical variables were compared using Fisher’s exact test or the χ2 test. Quantitative variables were expressed as mean with standard deviation (SD) or median with interquartile range. Differences between the groups were compared statistically using Student’s *t*-test for a normal distribution or the Mann–Whitney *U* test for a non-normal distribution. *P* values < 0.05 were considered statistically significant.

Cox proportional hazards regression models were used to evaluate the impact of pre-CKD on survival. HRs with 95% CIs were calculated after controlling for potential confounders, including patient demographics and clinicopathologic tumor variables. Additionally, we performed a Cox proportional hazards regression analysis using IPTW, which reweights affected and unaffected groups to emulate a propensity score-matched population [52] to evaluate the impact of preoperative CKD on prognosis. Variables included in the IPTW analysis were age, sex, ECOG PS, HTN, CVD, DM, NAC, urinary diversion, stage ≥pT3, LVI, and pN. We developed a prognostic factor-based risk stratification nomogram for 5-year PFS and OS with Cox proportional hazards regression analysis using the “rms” library in R. The *c*-index for predicting overall survival probability was calculated by an area under the curve using the receiver operating characteristic curve.

### Ethical standards

This study was performed in accordance with the ethical standards of the Declaration of Helsinki and approved by an ethics review board of Hirosaki University School of Medicine (authorization numbers; 2016–225 and 2015–258).

## SUPPLEMENTARY MATERIALS FIGURE




