# Supplementary material for: Preoperative chronic kidney disease predicts poor oncological outcomes after radical cystectomy in patients with muscle-invasive bladder cancer
Source: Oncotarget. 2017 May 29;8(37):61404–14. doi: 10.18632/oncotarget.18248 (PMC5617433; doi:10.18632/oncotarget.18248)
Supplement: Supplementary file 1 [file oncotarget-08-61404-s001.pdf]

Preoperative chronic kidney disease predicts poor oncological outcomes after radical cystectomy in patients with muscle-invasive bladder cancer

SUPPLEMENTARY MATERIALS

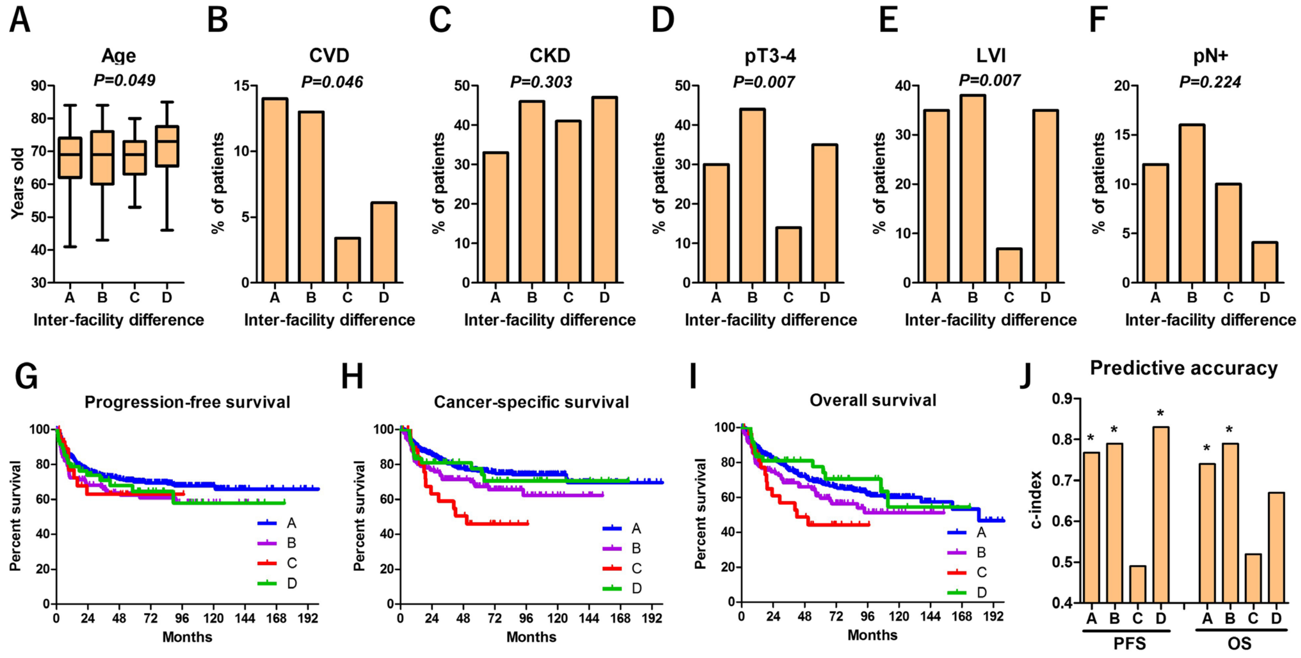

Supplementary Figure 1: Inter-facility difference in the present study. Inter-facility difference of patient backgrounds was compared among four hospitals (kruskal-wallis or χ^2 test). Age, prevalence of CVD, CKD, pT3-4, LVI, and pN+ were evaluated among the four hospitals. Age was significantly different among the four hospitals ($P = 0.049$, kruskal-wallis test) **(A)**. The prevalence of MIBC patients with CVD was significantly different among hospitals ($P = 0.046$, χ^2 test) **(B)**. The prevalence of MIBC patients with CKD was not significantly different ($P = 0.303$, χ^2 test) **(C)**. The number of patients with pT3-4 **(D)** and LVI **(E)** were significantly different ($P = 0.007$ and $P = 0.007$, respectively, χ^2 test), whereas those with pN+ were not differ among hospitals ($P = 0.224$, χ^2 test) **(F)**. Progression-free survival (PFS) **(G)**, cancer-specific survival (CSS) **(H)**, and overall survival (OS) **(I)** are shown. CSS and OS were shorter in patients in Hospital C than others. Internal validations of the four hospitals for nomograms of 5-year PFS and 5-year OS are shown **(J)** (*, $P < 0.001$). Predictive accuracy of 5-year nomograms were not significant in Hospital C (PFS: c-index = 0.49, $P = 0.925$, OS: c-index = 0.52, $P = 0.879$), and in Hospital D (OS: c-index = 0.67, $P = 0.077$).

Supplementary File 1:

See Supplementary File 1
